# Combined Effect of Deoxynivalenol (DON) and Porcine Circovirus Type 2 (Pcv2) on Inflammatory Cytokine mRNA Expression

**DOI:** 10.3390/toxins13060422

**Published:** 2021-06-13

**Authors:** Chao Gu, Xiuge Gao, Dawei Guo, Jiacai Wang, Qinghua Wu, Eugenie Nepovimova, Wenda Wu, Kamil Kuca

**Affiliations:** 1 MOE Joint International Research Laboratory of Animal Health and Food Safety, Engineering Center of Innovative Veterinary Drugs, College of Veterinary Medicine, Nanjing Agricultural University, Nanjing 210095, China; guchao0990@163.com (C.G.); vetgao@njau.edu.cn (X.G.); gdawei0123@njau.edu.cn (D.G.); 2 Shandong Vocational Animal Science and Veterinary College, 88 Shengli East Street, Weifang 261061, China; sdmyjcw@163.com; 3 Department of Chemistry, Faculty of Science, University of Hradec Králové, Rokitanského 62, 500 03 Hradec Kralove, Czech Republic; wqh212@yangtzeu.edu.cn (Q.W.); eugenie.nepovimova@uhk.cz (E.N.); 4 College of Life Science, Yangtze University, Jingzhou 434025, China; 5 Biomedical Research Center, University Hospital Hradec Kralove, 500 03 Hradec Kralove, Czech Republic

**Keywords:** deoxynivalenol, PCV2, IL-1β, IL-6, MAPK

## Abstract

A host’s immune system can be invaded by mycotoxin deoxynivalenol (DON) poisoning and porcine circovirus type 2 (PCV2) infections, which affect the host’s natural immune function. Pro-inflammatory cytokines, IL-1β and IL-6, are important regulators in the process of natural immune response, which participate in inflammatory response and enhance immune-mediated tissue damage. Preliminary studies have shown that DON promotes PCV2 infection by activating the MAPK signaling pathway. Here, we explored whether the mRNA expression of IL-1β and IL-6, induced by the combination of DON and PCV2, would depend on the MAPK signaling pathway. Specific pharmacological antagonists U0126, SP600125 and SB203580, were used to inhibit the activities of ERK, JNK and p38 in the MAPK signaling pathway, respectively. Then, the mRNA expression of IL-1β and IL-6 in PK-15 cells was detected to explore the effect of the MAPK signaling pathway on IL-1β and IL-6 mRNA induced by DON and PCV2. The results showed that PK-15 cells treated with DON or PCV2 induced the mRNA expression of IL-1β and IL-6 in a time- and dose-dependent manner. The combination of DON and PCV2 has an additive effect on inducing the mRNA expression of IL-1β and IL-6. Additionally, both DON and PCV2 could induce the mRNA expression of IL-1β and IL-6 via the ERK and the p38 MAPK signal pathways, while PCV2 could induce it via the JNK signal pathway. Taken together, our results suggest that MAPKs play a contributory role in IL-1β and IL-6 mRNA expression when induced by both DON and PCV2.

## 1. Introduction

Trichothecene mycotoxins, the secondary metabolites produced by fungi such as *Fusarium* and *Trichothecium*, are widely distributed across the world [1]. Since these toxins have been linked to human and animal toxicoses, their presence in global food commodities and feedstuffs is a matter of considerable public health concern [2]. Deoxynivalenol (DON, Vomitoxin), the most abundant trichothecene mycotoxin associated with *Fusarium* head blight (FHB), can survive processing and persist into the food chain. In humans and animals, DON has been associated with a series of adverse effects including anorexia, vomiting, growth retardation, diarrhea, neuroendocrine changes, gastrointestinal inflammation, and immunosuppression [3]. The immuno-toxic effects induced by DON is of particular concern from the perspective of human and animal health. According to the timing and dose of exposure, DON has the potential to elicit either an immunosuppressive response or immune stress [4]. Our previous studies indicate that exposure to DON induces the overexpression of proinflammatory cytokines, such as IL-1β and IL-6, in the plasma and organs of mouse [5,6]. 

Cytokines are a class of small molecular proteins with a size of 5-20kDa. They play a role in a wide range of biological activities and in a variety of life activities inside the body [7]. The expression of proinflammatory cytokines is aberrantly upregulated for the activation of the innate immune system, leading to immune stress, which can cause physiological and immune function impairment [8]. A large number of studies have shown that proinflammatory cytokines including IL-1β and IL-6 can cause anorexia, daily weight decrease, a decrease in immunity causing inflammation, and an increased likelihood of other diseases occurring [9,10]. This is similar to DON causing animal refusal, malnutrition, and secondary infection with other pathogens [11]. In addition to the capacity of DON to upregulate proinflammatory cytokines in vivo, some studies have found that DON also increased the mRNA and protein expression of IL-1β and IL-6 in human peripheral blood mononuclear cells, human monocyte cell lines and mouse macrophage cell lines [12,13,14]. DON may target phagocytes to produce immunotoxicity. 

Porcine circovirus (PCV), first discovered in 1974 as a contaminant of a continuous porcine kidney cell line (PK-15), is classified in the genus *Circovirus* of the family *Circoviridae* [15]. Two genotypes of PCV have been identified. PCV type 1 (PCV1) is known to be nonpathogenic to pigs. PCV type 2 (PCV2) is a DNA virus that can severely damage the respiratory, digestive and nervous systems of pigs of all ages, among which piglets are the most sensitive [16,17]. Pigs infected with PCV2 have multi-system inflammation, indicating disordered expression of proinflammatory cytokines. Some studies have reported that PCV2 infects porcine alveolar macrophages (PAM) to activate NF-κB and induce IL-1β overexpression [18,19]. Another study showed that the expression of IL-1β and IL-6 mRNA in piglets suffering from postweaning multisystemic wasting syndrome (PMWS), caused by PCV2, was significantly up-regulated [20]. However, the reasons why PCV2 infection causes the production and/or secretion of cytokines and the imbalance of cytokines are not completely clear, and the regulatory mechanism involved is still unclear.

As an important signal pathway that transfers signals from the cell surface to the nucleus, the mitogen-activated protein kinase (MAPK) pathway includes extracellular signal regulated protein kinase 1 and 2 (ERK 1/2), p54 and p46 c-Jun N-terminal kinase 1 and 2 (JNK 1/2), and p38 [21]. After being subjected to extracellular stimuli such as toxins and pathogens, MAPK can be sequentially activated and contribute to various pathological and physiological processes such as inflammatory response, stress adaptation, cell growth and differentiation [22]. The MAPK pathway is also closely related to DON poisoning and PCV2 infection [23,24]. However, the role of MAPK signaling pathway in the induction of proinflammatory cytokines such as IL-1β and IL-6 by DON and PCV2 in PK-15 cell remains unclear and requires further study.

In this study, we evaluated the effect of MAPK on the expression of IL-1β and IL-6 cytokines that mRNA induced by DON and PCV2. We found that the MAPK pathway can up-regulate mRNA levels of relative cytokine in PK-15 cells after DON and/or PCV2 treatment, leading to changes in inflammation and immune function. Our findings will provide a new perspective to advance the understanding of the mechanisms of DON poisoning and PCV2 infection, as well as providing new ideas for the prevention and control of both DON and PCV2.

## 2. Results

### 2.1. DON Exposure Induces Elevations in IL-1β and IL-6 mRNA

**Figure 1:**
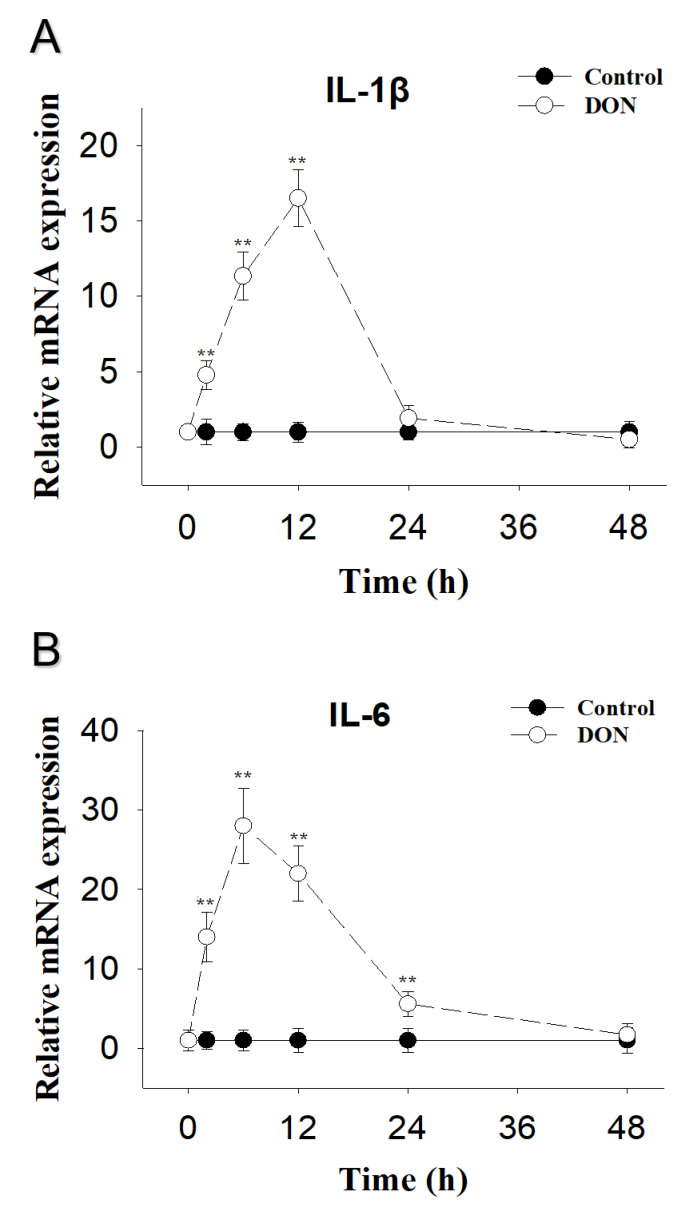
DON-induced cytokine IL-1β and IL-6 mRNA upregulation in PK-15 cells. qRT-PCR were performed to analyze the mRNA expression of IL-1β (**A**) and IL-6 (**B**). Data are mean ± SEM (n = 3). Symbol ** *p* < 0.01.

DON-induced PK-15 cells IL-1β mRNA were elevated at 2 h, reached peak concentrations at 12 h and returned to basal level at 24 h post-exposure (Figure 1A). IL-6 mRNA was upregulated and reached peak concentrations at 6 h, and were still markedly raised at 24 h, but returned to basal level at 48 h post-exposure (Figure 1B).

**Figure 2:**
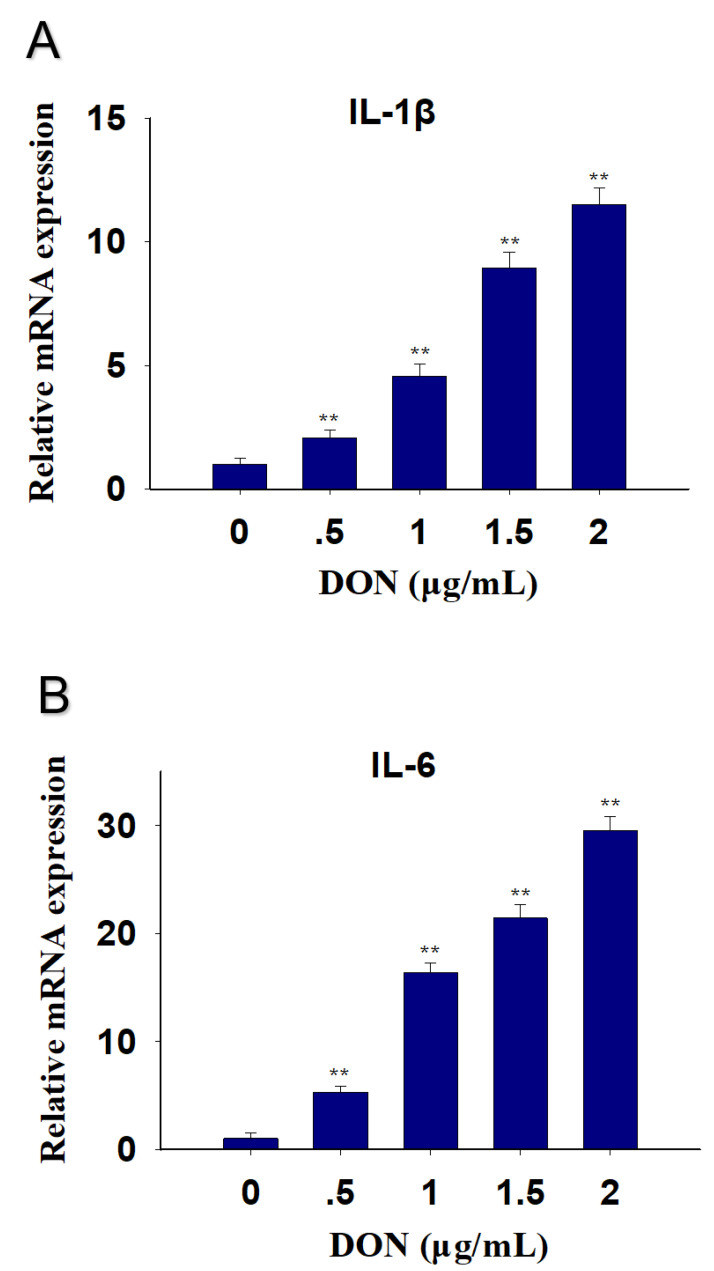
Different concentrations of DON-induced cytokine IL-1β and IL-6 mRNA upregulation in PK-15 cells. qRT-PCR were performed to analyze the mRNA expression of IL-1β (**A**) and IL-6 (**B**). Data are mean ± SEM (n = 3). Symbol ** *p* < 0.01.

DON in 0.5, 1, 1.5 and 2 μg/mL upregulation PK-15 cells IL-1β mRNA by 3-, 5-, 10- and 13-fold at 12 h, respectively (Figure 2A). IL-6 mRNA expression was elevated by 6-, 17-, 22- and 28-fold (Figure 2B).

### 2.2. PCV2 Infection Induces Elevations in IL-1β and IL-6 mRNA

**Figure 3:**
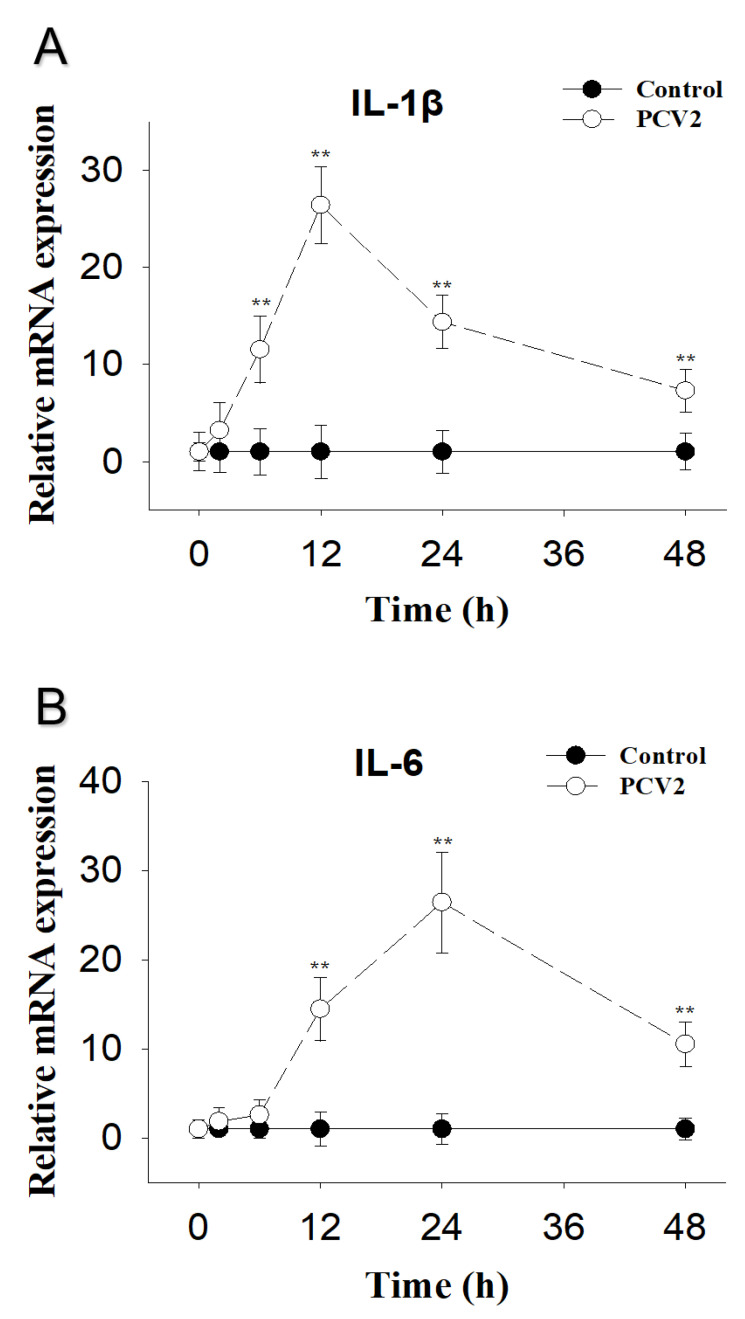
PCV2-induced cytokine IL-1β and IL-6 mRNA upregulation in PK-15 Cells. qRT-PCR were performed to analyze the mRNA expression of IL-1β (**A**) and IL-6 (**B**). Data are mean ± SEM (n = 3). Symbol ** *p* < 0.01.

PCV2-induced PK-15 cells IL-1β mRNA were elevated at 6 h, reached peak concentrations at 12 h, and were still markedly raised at 48 h post-exposure (Figure 3A). IL-6 mRNA was upregulated at 12 h, reached peak concentrations at 24 h, and was still markedly raised at 48 h post-exposure (Figure 3B).

**Figure 4:**
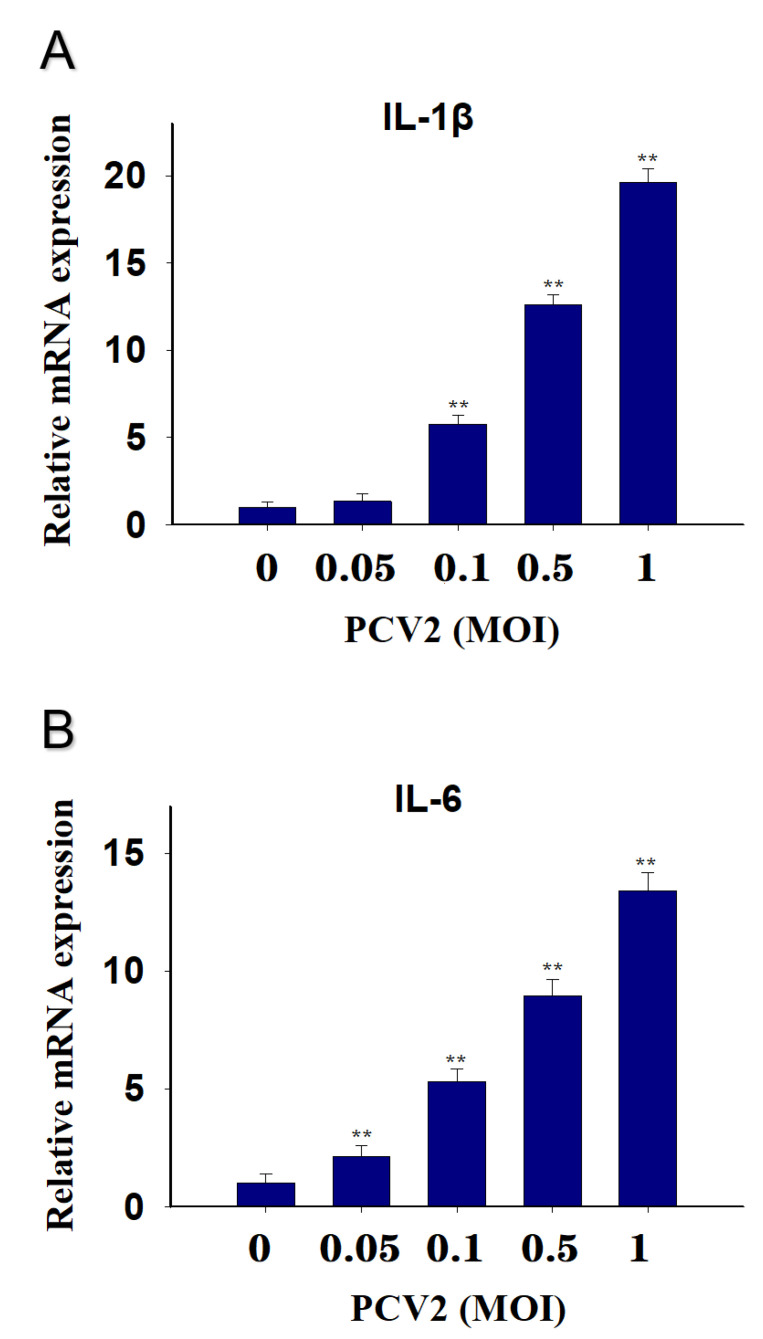
Different MOI of PCV2 induced cytokine IL-1β and IL-6 mRNA upregulation in PK-15 Cells. qRT-PCR were performed to analyze the mRNA expression of IL-1β (**A**) and IL-6 (**B**). Data are mean ± SEM (n = 3). Symbol ** *p* < 0.01.

PCV2 in 0.1, 0.5 and 1 MOI upregulation PK-15 cells IL-1β mRNA by 6-, 14-, and 19-fold at 12 h, respectively, while 0.05 MOI had no effect (Figure 4A). IL-6 mRNA expression was elevated by 3-, 6-, 9- and 14-fold, respectively (Figure 4B).

### 2.3. Combined Effect of DON and PCV2 Induces the Expression of IL-1β and IL-6 mRNA 

**Figure 5:**
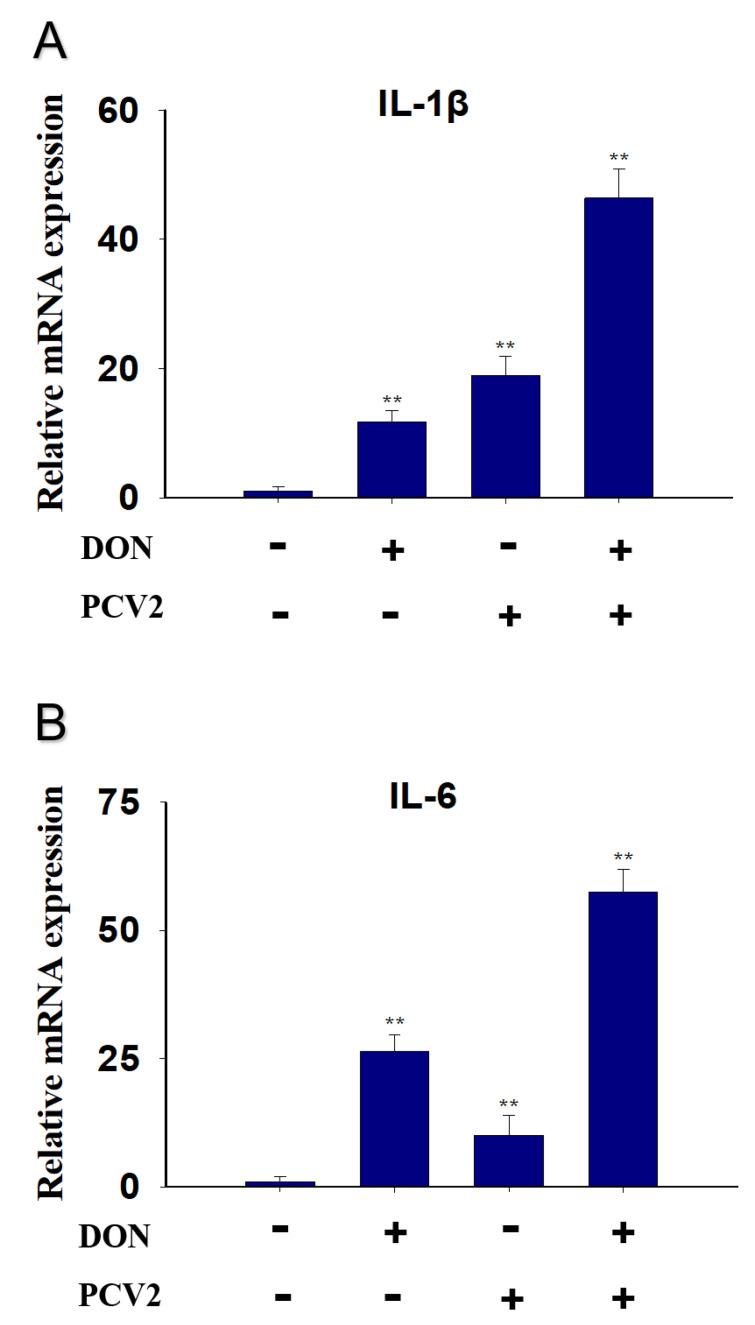
Combined effect of DON and PCV2 induced cytokine IL-1β and IL-6 mRNA upregulation in PK-15 Cells. qRT-PCR were performed to analyze the mRNA expression of IL-1β (**A**) and IL-6 (**B**). Data are mean ± SEM (n = 3). Symbol ** *p* < 0.01.

IL-1β mRNA expression was elevated by DON (10-fold), PCV2 (20-fold) and the combined effect of DON and PCV2 (45-fold) (Figure 5A). As for IL-6, the mRNA expression was markedly increased by DON (28-fold), PCV2 (10-fold) and the combined effect of DON and PCV2 (56-fold) (Figure 5B).

### 2.4. DON and PCV2 Induce the Expression of IL-1β and IL-6 mRNA via ERK Signaling Pathway 

**Figure 6:**
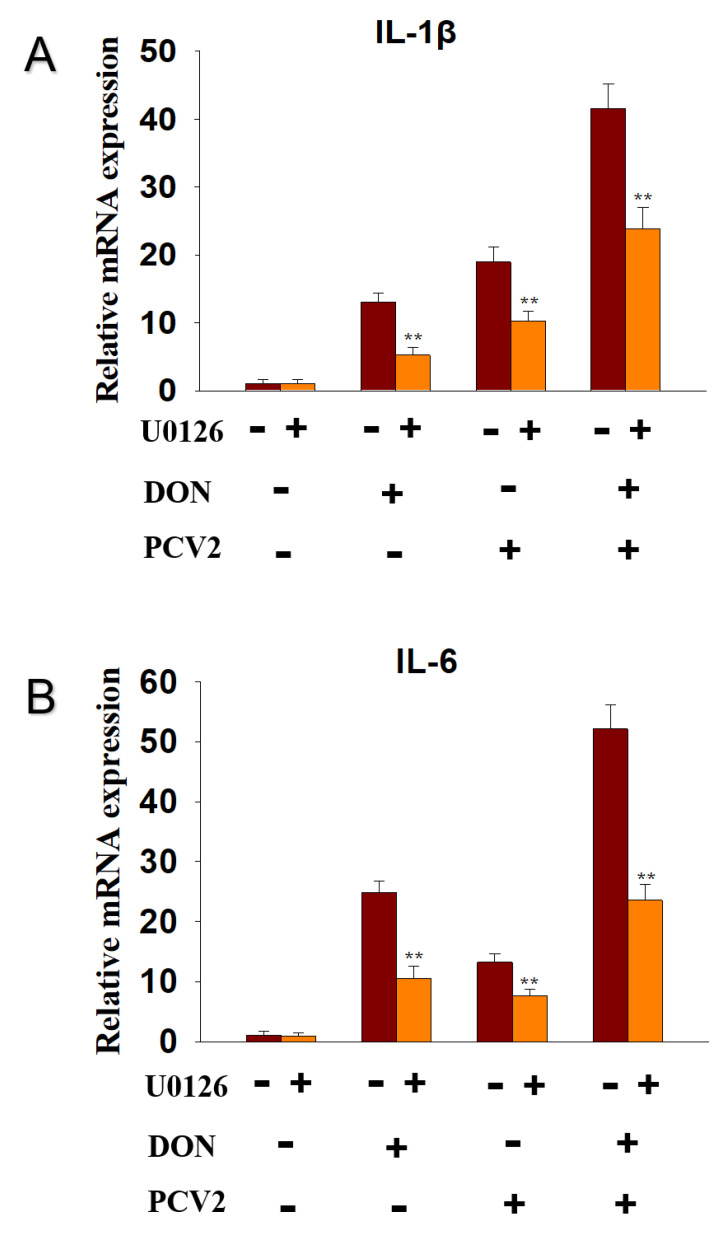
ERK participated in DON and PCV2-induced cytokine IL-1β and IL-6 mRNA upregulation in PK-15 cells. qRT-PCR were performed to analyze the mRNA expression of IL-1β (**A**) and IL-6 (**B**). Data are mean ± SEM (n = 3). Symbol ** *p* < 0.01.

To explore whether the ERK signaling pathway participated in the DON and PCV2-induced IL-1β and IL-6 mRNA expression, the inhibitor of p-ERK, U0126, was supplied. The data showed that U0126 decreased IL-1β mRNA expression in the DON group from 12-fold to 5-fold, in the PCV2 group from 20-fold to 10-fold, and in the DON+PCV2 group from 41-fold to 22-fold (Figure 6A). U0126 decreased IL-6 mRNA expression in the DON group from 25-fold to 10-fold, in the PCV2 group from 13-fold to 8-fold, and in the DON+PCV2 group from 52-fold to 22-fold (Figure 6B).

### 2.5. PCV2 Induces the Expression of IL-1β and IL-6 mRNA via JNK Signaling Pathway 

**Figure 7:**
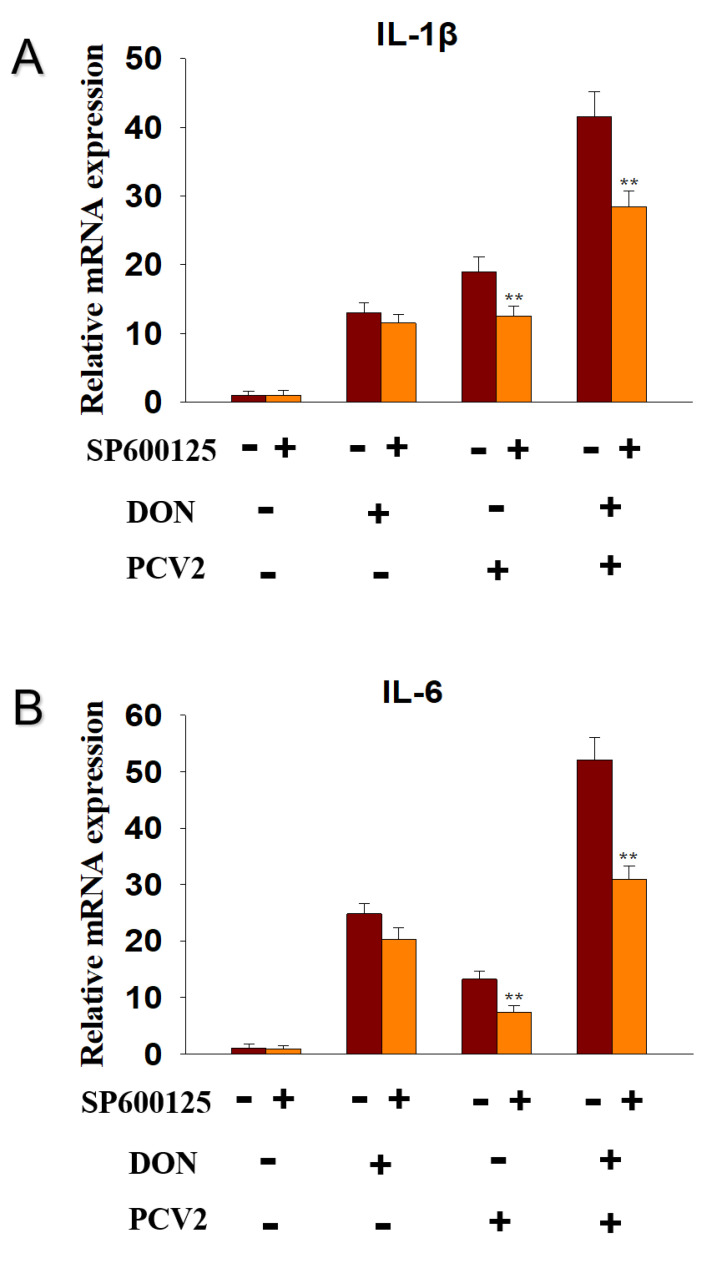
JNK participated in PCV2-induced cytokine IL-1β and IL-6 mRNA upregulation in PK-15 Cells. qRT-PCR were performed to analyze the mRNA expression of IL-1β (**A**) and IL-6 (**B**). Data are mean ± SEM (n = 3). Symbol ** *p* < 0.01.

To explore whether JNK signaling pathway participated in the DON and PCV2-induced IL-1β and IL-6 mRNA expression, the inhibitor of p-JNK, SP600125, was supplied. The data showed that SP600125 decreased IL-1β mRNA expression in the PCV2 group from 20-fold to 11-fold and in the DON+PCV2 group from 41-fold to 28-fold, while the DON group had no effect (Figure 7A). SP600125 decreased IL-6 mRNA expression in the PCV2 group from 12-fold to 7-fold and in the DON+PCV2 group from 52-fold to 30-fold, while the DON group had no effect (Figure 7B).

### 2.6. DON and PCV2 Induce the Expression of IL-1β and IL-6 mRNA via p38 Signaling Pathway 

**Figure 8:**
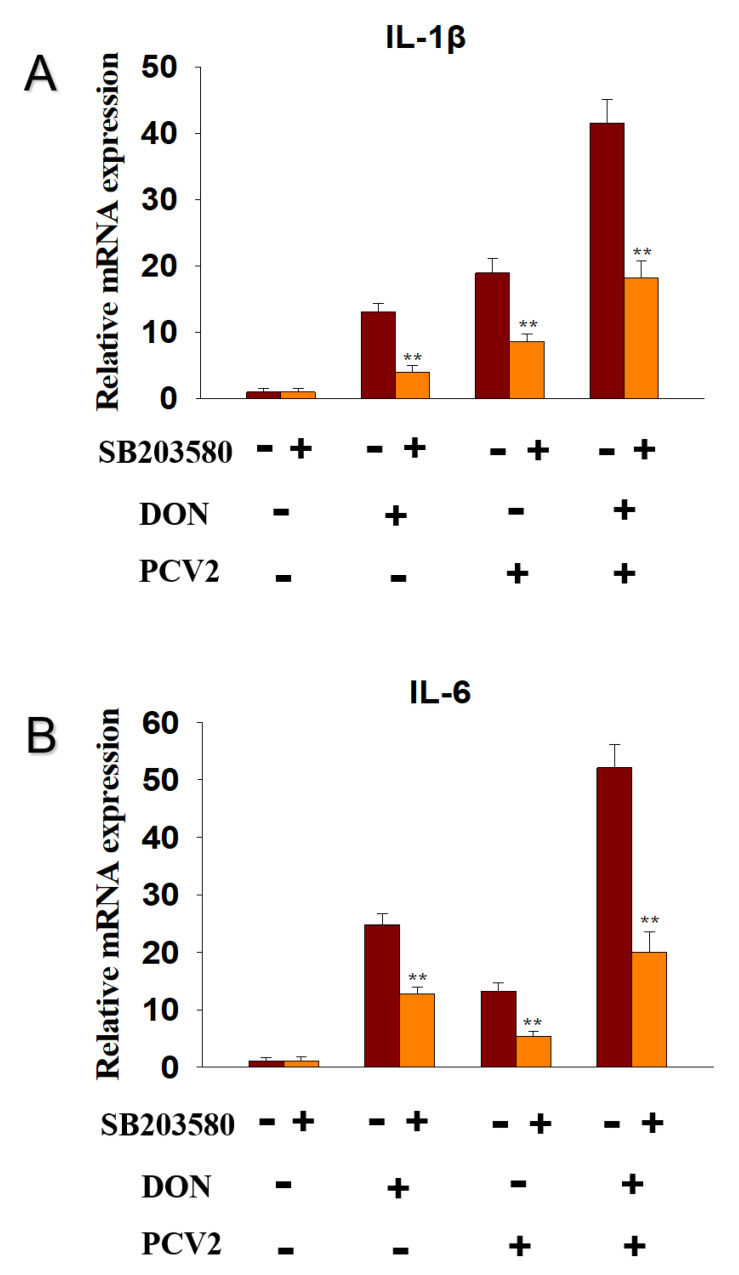
p38 participated in DON and PCV2-induced cytokine IL-1β and IL-6 mRNA upregulation in PK-15 cells. qRT-PCR were performed to analyze the mRNA expression of IL-1β (**A**) and IL-6 (**B**). Data are mean ± SEM (n = 3). Symbol ** *p* < 0.01.

To explore whether ERK signaling pathway participated in the DON and PCV2-induced IL-1β and IL-6 mRNA expression, the inhibitor of p-p38, SB203580, was supplied. The data showed that SB203580 decreased IL-1β mRNA expression in the DON group from 13-fold to 5-fold, in the PCV2 group from 19-fold to 9-fold, and in the DON+PCV2 group from 41-fold to 18-fold (Figure 8A). SB203580 decreased IL-6 mRNA expression in the DON group from 24-fold to 11-fold, in the PCV2 group from 12-fold to 5-fold, and in the DON+PCV2 group from 52-fold to 20-fold (Figure 8B).

## 3. Discussion

Mycotoxins are widespread in the environment and coexist alongside other pathogens such as virus and bacteria. To a certain extent, mycotoxins enhance the pathogenicity of other pathogens [25,26,27,28]. DON has the potential to evoke a wide spectrum of patho-physiological effects that are partly attributable to a ribo-toxic stress-mediated cytokine storm [29]. With respect to PCV2 infection, the immune injury is always accompanied by a change in proinflammatory cytokine expression, including IL-1β and IL-6 [30]. In this study, we focused on the role of the MAPK signaling pathway in the mRNA expression of IL-1β and IL-6, induced by DON and PCV2. Several key findings were evident and demonstrate that (1) DON and PCV2 induced the mRNA expression of IL-1β and IL-6 in a time- and dose-dependent manner, respectively. (2) The combination of DON and PCV2 has an additive effect on the induction of the mRNA expression of IL-1β and IL-6 in the PK-15 cell. (3) DON induced the mRNA expression of IL-1β and IL-6 via the ERK and p38 MAPK signaling pathways and (4) PCV2 induced the mRNA expression of IL-1β and IL-6 via the ERK, JNK and p38 MAPK signaling pathways.

The dose response of DON-induced IL-1β and IL-6 mRNA expression suggested that the mRNA expression of these two cytokines increased after PK-15 cells were challenged with different concentrations of DON at 0.5, 1, 1.5, and 2 μg/mL. Furthermore, the kinetics of IL-1β and IL-6 mRNA responses to DON indicated that upregulation of these genes was maximal at 12 h and 6 h, respectively. These findings are consistent with several in vitro studies by Pestka and co-workers [31,32]. For instance, from 100 to 1000 ng/mL of DON significantly increased production of IL-6 from 3 h to 24 h in U-937 cells [31]. Robustly elevated IL-1β and IL-6 intracellular protein and mRNA expression was also observed in peripheral blood mono-nuclear cells treated with DON at 500 ng/mL [32]. DON’s in vitro effects on IL-1β and IL-6 can be reproduced in a mouse, a commonly used model of the human immune system. DON has the capacity to induce IL-1β and IL-6 mRNAs not only in the plasma, but also in organs such as the spleen, liver, lung, kidney, small intestine and brain [33,34]. Eventually, both cytokines were returned to basal levels in this study. A possible reason for the decreased cytokines response might be mRNA degradation caused by reduced MAPK activation [35,36].

Similarly, PCV2 was shown to induce the expression of IL-1β and IL-6 at the transcriptional level in PK-15 cells through time- and dose-dependent manners. Consistent with this finding, PCV2 was reported to increase IL-1β production in porcine alveolar macrophages, and the changes in cytokine expression are related to the TLR-MyD88-NF-κB signaling pathway [19]. In PK-15 cells, PCV2 was reported to elevate IL-6 production via suppressor of cytokine signaling 3 [37]. In addition to in vitro studies, the levels of IL-1β and IL-6 in both serum and spleen were significantly upregulated after PCV2 infection in the mouse [30]. Proinflammatory cytokines are important factors for the elimination of invading pathogens [38]. Excessive release of proinflammatory cytokines can lead to undesired tissue lesions and decrease the body’s immunity to other pathogen infections [39]. Therefore, controlling the inflammatory response is crucial. However, environmental factors, such as toxins, can interfere with this control.

This is the first report to demonstrate that co-treatment with DON and PCV2 in PK-15 cells can enhance the up-regulation effect of IL-1β and IL-6 mRNA with an additive effect. The possible reason may be related to the ability of the mycotoxin to promote virus replication, leading to an increase in cytokines expression. Qian et al indicated that mycotoxin ochratoxin A had the capacity to induce PCV2 replication promotion in PK-15 cells [40]. The molecular mechanisms of this effect are associated with ochratoxin A-induced autophagy involving in AKT/mTOR and ERK1/2 MAPK signaling pathway [41]. DON significantly promoted the replication of porcine epidemic diarrhea virus in IPEC-J2 cells, along with the induction of a complete autophagy triggered by p38 MAPK signaling pathway [42]. Our preliminary data also indicate that DON promotes PCV2 infection by activating the MAPK signaling pathway. However, the underlying mechanism of this effect still requires further research to substantiate such findings.

The results presented here demonstrate that ERK and p38 MAPK participate in DON-induced IL-1β and IL-6 mRNA up-regulation in PK-15 cells. PCV2, however, induced IL-1β and IL-6 mRNA up-regulation via the ERK, JNK and p38 MAPK signaling pathways. Some studies have found that the expression of cytokine genes is caused by DON-mediated rRNA perforation and the induction of damage-related molecular patterns (DAMPs) by ribosomal-related stress kinases [43,44]. The latter can activate members of the MAPK family, which mediate transcription factor activation and mRNA stabilization, and lead to an increased expression of the pro-inflammatory gene mRNA and ultimately, protein [14]. He and co-workers found that the ability of DON to change the translation and expression of inflammation-related genes is mainly driven by selective transcription and mRNA stabilization through ERK and p38 MAPK signaling pathways [13]. On the other hand, studies have also shown that activation of the ERK, JNK and p38 MAPK signaling pathways contribute to the promotion PCV2 infection [30,37]. These viewpoints are consistent with the findings in our study. 

## 4. Conclusions

Treatment of PK-15 cells with DON or PCV2 can induce the expression of IL-1β and IL-6 mRNA; this is both time-dependent and dose-dependent. Furthermore, the combined effect of DON and PCV2 could increase the expression of IL-1β and IL-6 mRNA. The expression of IL-1β and IL-6 mRNA induced by DON is dependent on the ERK and p38 MAPK signaling pathways, while the expression of IL-1β and IL-6 mRNA induced by PCV2 depends on the ERK, JNK, and p38 MAPK signaling pathways.

## 5. Materials and Methods

### 5.1. Toxin and Virus

Deoxynivalenol (DON) were purchased from Sigma-Aldrich (Shanghai, China). PCV2 strains were kindly donated by the Laboratory of Infectious Disease, Department of Prevention Veterinary Medicine, Nanjing Agricultural University. It was isolated and sequenced from the kidneys of piglets, naturally infected with multiple system failure syndrome of weaned piglets, and stored at −80 °C.

### 5.2. Cell Cultures and Virus Cultures

Porcine kidney cell (PK-15) cell line (without PCV contamination) was kindly donated by the Laboratory of Internal Veterinary Medicine, Department of Clinical Veterinary Medicine, Nanjing Agricultural University. All cells were cultured in DMEM (Gibco, Shanghai, China) medium with 10% newborn calf serum (Gibco, Shanghai, China) and 1% penicillin, at 37 °C and 5% carbon dioxide.

PCV2 was amplified using PK-15 cells. The cytopathic effect (CPE) was observed and PCV2 was detected by the indirect immunofluorescence assay in inoculated PK-15 cells. The viral titers were determined to be 10^6.1^ TCID50/0.1 mL, using the Reed–Muench assay.

### 5.3. Experimental Design

Three specific inhibitors U0126, SP600125 and SB203580 (MedChemExpress, Shanghai, China) were added in the PK-15 cells to block ERK, JNK and p38 MAPK signaling pathways, respectively. Then, PK-15 cells were treated with DON or PCV2. Total cell RNA was extracted, then an ultra-micro nucleic acid protein analyzer was used to determine OD260/OD280 value and detect RNA quality. qRT-PCR was used to detect the expression of IL-1β and IL-6 mRNA.

### 5.4. Quantative Real-Time PCR (qRT-PCR) Analysis

Total RNA was isolated from PK-15 cells using TRIzol Reagent (Takara, Dalian, China). cDNA was obtained by reverse transcription using a cDNA transcription kit (Takara, Dalian, China). qRT-PCR was performed using SYBR Premix Ex Taq™ (Takara, Dalian, China) and the primers are shown as IL-1β (F: 5′-TACCTCTTGGAGGCACAAAGG-3′ and R:5′-CTTCCTTGGCAGGTTCAGGT A-3′), IL-6 (F: 5′-AGCAAGGAGGTACTGGCAGA-3′ and R: 5′-CAGGGTCTGGATCAGTGCTT-3′) and GAPDH (F: 5′-CGTCAAGCTCATTTCCTGGT-3′ and R: 5′-TGGGATGGAAACTGGAAGTC-3′). Fold changes in cytokines were determined using 2^(−ΔΔCt)^ method and gene expression levels were normalized to GAPDH [45]. qRT-PCR was performed using the ABI PRISM 7900HT Real-Time PCR System.

### 5.5. Statistical Analyses

Statistical analyses were performed using GraphPad Prism 8.0 Software (GraphPad Software, Inc., San Diego, CA, USA). Data for each assay were analyzed with one-way analysis of variance (ANOVA) or two-way ANOVA. Data were expressed as the mean ± SEM. Statistical significance was set at *p* < 0.05.

## Data Availability

The data presented in this study are available upon request to the corresponding author.
